# Precision, integrative medicine for pain management in sickle cell disease

**DOI:** 10.3389/fpain.2023.1279361

**Published:** 2023-11-09

**Authors:** Wally R. Smith, Cecelia R. Valrie, Cheedy Jaja, Martha O. Kenney

**Affiliations:** ^1^ Division of General Internal Medicine, Virginia Commonwealth University, Richmond, VA, United States; ^2^ Department of Psychology, Virginia Commonwealth University, Richmond, VA, United States; ^3^ College of Nursing, University of South Florida School of Nursing, Tampa, FL, United States; ^4^ Department of Anesthesiology, Duke University, Durham, NC, United States

**Keywords:** pain, sickle cell disease, integrative medicine, precision medicine, pain neuromatrix

## Abstract

Sickle cell disease (SCD) is a prevalent and complex inherited pain disorder that can manifest as acute vaso-occlusive crises (VOC) and/or chronic pain. Despite their known risks, opioids are often prescribed routinely and indiscriminately in managing SCD pain, because it is so often severe and debilitating. Integrative medicine strategies, particularly non-opioid therapies, hold promise in safe and effective management of SCD pain. However, the lack of evidence-based methods for managing SCD pain hinders the widespread implementation of non-opioid therapies. In this review, we acknowledge that implementing personalized pain treatment strategies in SCD, which is a guideline-recommended strategy, is currently fraught with limitations. The full implementation of pharmacological and biobehavioral pain approaches targeting mechanistic pain pathways faces challenges due to limited knowledge and limited financial and personnel support. We recommend personalized medicine, pharmacogenomics, and integrative medicine as aspirational strategies for improving pain care in SCD. As an organizing model that is a comprehensive framework for classifying pain subphenotypes and mechanisms in SCD, and for guiding selection of specific strategies, we present evidence updating pain research pioneer Richard Melzack’s neuromatrix theory of pain. We advocate for using the updated neuromatrix model to subphenotype individuals with SCD, to better select personalized multimodal treatment strategies, and to identify research gaps fruitful for exploration. We present a fairly complete list of currently used pharmacologic and non-pharmacologic SCD pain therapies, classified by their mechanism of action and by their hypothesized targets in the updated neuromatrix model.

## 1. Introduction

Most pain, including sickle cell disease (SCD) pain, is managed at home without professional supervision (1–4). And when individuals with pain present to clinicians, they are most often treated with palliative approaches. Drugs such as non-steroidal anti-inflammatory drugs, antidepressants, anticonvulsants, local anesthetics, muscle relaxants, and antianxiety medicines may be used, as may non-pharmacological interventions (5). For SCD pain in western countries, one of the most common palliative therapies used is opioid therapy (6, 7). Though expert guidelines advocate for opioid-sparing approaches, employing multimodal combinations of therapy (8, 9), usually administered by a multidisciplinary team (10), they are based mostly on expert consensus, and often poorly adhered to (11, 12). Treatment can be frustrating for clinicians trained to apply scientific and mechanistic principles to the practice of medicine.

### 1.1. Precision medicine and pharmacogenomics in SCD

Precision medicine (13) has been defined as “the tailoring of medical treatment to the individual characteristics of each patient…to classify individuals into subpopulations that differ in their susceptibility to a particular disease or their response to a specific treatment.” (14) Applied to pain, precision medicine involves phenotyping pain patterns and operative mechanisms, genetics, and expressions of pain in each individual; then creating or revising an individualized pain management plan for all phases of pain care based on observed phenotypic and mechanistic features. Precision medicine acknowledges individuals’ unique differences in disease susceptibility and drug metabolism, depending on variations in their genetic makeup.

Because of the intricate interplay between genetics and pain expression and analgesic response, pharmacogenomics, a precision medicine modality, can potentially play a crucial role in safe and effective sickle cell disease pain management (15). Pharmacogenomics is the genomic profiling of patients for genetic variants that clinically modify the tolerability and desired effect of specific medications (16). For instance, the cytochrome *P450* (*CYP)* genes variants have an impact on the metabolism of multiple medications across analgesic drug classes. Patients who poorly metabolize certain analgesic drugs may face mild, moderate, or even severe toxicities, requiring acute care admission or even hospitalization (17). Further, severe or even mild adverse events may lower adherence, and genetic variance may be associated with reduced drug efficacy and increased medication wastage.

Pharmacogenomics-guided prescribing guidelines for approximately 80 drugs have been developed by international collaborative groups such as the Clinical Pharmacogenetics Implementation Consortium (CPIC) and the Dutch Pharmacogenetics Working Group (DPWG) (18). Almost two-thirds of these actionable drug-gene associations involve drug-metabolizing enzyme genes, about 80% of which encode cytochrome *P450 (CYP)* enzymes (19). Preemptive pharmacogenetic testing to predict a patient’s metabolic response can facilitate individualized prescribing (20), but genotype-guided dosing practice is not readily available in SCD care (21).

### 1.2. Integrative health

Integrative health “includes whole person health, that is, empowering individuals, families, communities, and populations to improve their health in multiple interconnected domains: biological, behavioral, social, and environmental.” Modes of integrative healthcare have received increasing attention and trials among individuals with SCD, especially for pain (22). Integrative approaches come in two subtypes. If a non-mainstream practice is used *together with* conventional medicine, it is considered “complementary.” If a non-mainstream practice is used *in place of* conventional medicine, it is considered “alternative.” Complementary approaches are currently the most ones used to treat chronic pain (23).

### 1.3. Models and mechanisms of pain

Precision medicine and integrative health both evoke a transcendent causal and treatment model of pain to replace the long-held and accepted Cartesian pain model (24). The Cartesian model views pain purely as a biomarker of tissue damage or inflammation that is detected by the peripheral nervous system. The role of = ascending sensory fibers, especially the first-order neurons in the spinothalamic tract, the dorsal root ganglia, and the second-order ascending fibers, is to transmit pain signals unmodulated to the central nervous system (CNS) (25, 26). The role of the CNS is only to receive and process these unmodulated pain signals, leading to passive perception of pain, and some kind of reaction. In contrast, a transcendent pain model consistent with the practice of precision medicine and integrative health must explain the observed pain phenotypes, pain mechanisms, and pain findings which aren’t explained by the Cartesian model. Decades ago, Richard Melzack proposed such a model, the Neuromatrix theory of pain (27, 28). It theorizes that multiple parts of the nervous system– the “body-self neuromatrix”—work together to generate pain, and that *pain can be produced independently of peripheral sensory input* (29). It is consistent with Engel’s general biopsychosocial model (30–33), because it theorizes environmental as well as internal biological influences on pain. The neuromatrix is genetically predetermined (34), but is biologically responsive to stimuli. Further, pain responses in the neuromatrix may be so altered by the environment that they no longer require a synchronous environmental stimulus, such as in phantom limb pain (35).

**Figure 1:**
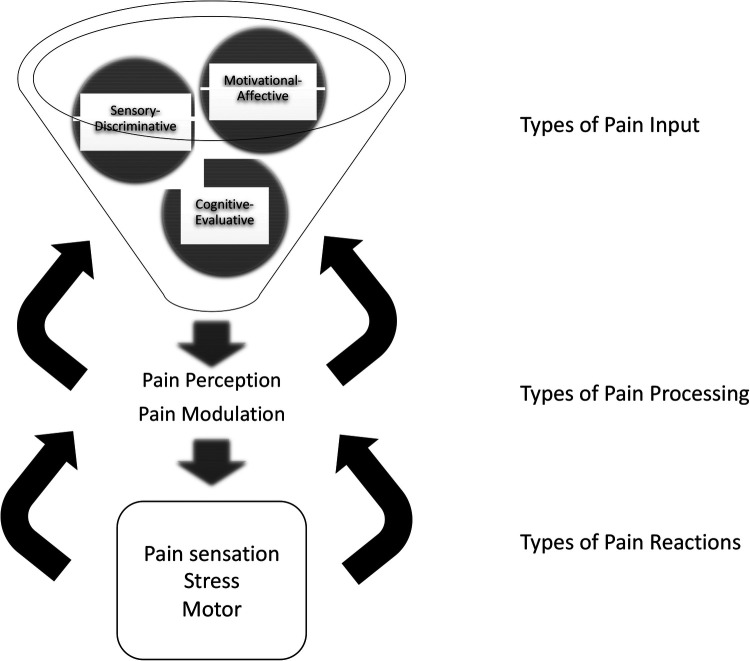
Updated neuromatrix model of pain with components. Three types of input, sensory-discriminative, cognitive-evaluative, and motivational-affective, integrate into pain processors. Processing includes perception in midbrain and thalamic brain structures and a network of connections between them, and modulation by these connections and by ascending and descending fibers. Three types of resulting pain reactions include pain perception/sensation, motor function, and stress. Feedback modulates every stage: reactions modulate processing, and processing modulates input signals. Pain intervention targets are suggested by each component. (Adapted from Melzack and numerous authors).

Since Melzack’s theory was proposed, animal models, human imaging studies (36) including functional magnetic resonance imaging studies (37), and even invasive brain electrode studies (38) have led to a consensus that a distributed, anatomical neuromatrix is indeed responsible for receipt and processing of a variety of pain signals (Figure 1). For acute pain, this network consists not only of the ascending pain fibers, but also of the primary and secondary somatosensory, insular, anterior cingulate, and prefrontal cortices and the thalamus (36). But more important than these multiple structures is the network of connections between the structures. Connections in this network are constantly shifting (39, 40). We hypothesize, but are still unsure how, shifts in these connections over time can cause a transformation from acute pain to chronic pain. However, evidence is accumulating to show that this same pain neuromatrix, this network, is operative in patients with SCD (41–44).

The updated pain neuromatrix suggests multipe targets for pain therapy for SCD. Interventions may be directed either at three types of inputs, i.e., sensory-discriminative, cognitive-evaluative, and motivational-affective inputs. Or interventions may be directed at pain processing, i.e., interventions may alter how pain signals are integrated, modulated, and/or passed on in the pain neuromatrix.

Regarding inputs, sensory-discriminative inputs consist of cutaneous, musculoskeletal, visceral, visual, and other sensory inputs, which define the location and quality of pain perception. Cognitive-evaluative inputs provide interpretation or meaning for the pain experience. This includes factors, such as memories of past experiences, cultural understanding of pain, pain rumination, pain catastrophizing (45), and somatization of pain. Motivational-affective inputs add an emotion or feeling to the pain experience. They ask, “what should I feel and/or do about my pain?” The limbic system and its associated regulation of autonomic and endocrine responses, including in the gut (46) provides motivational-affective input. Its role in SCD pain requires further exploration (47, 48); however, many studies report comorbid depression and anxiety (49), commonly seen in many pain states.

Regarding pain processing of these types of pain inputs, integration into a summary pain perception, as well as modulation of the inputs, occurs in the network of midbrain and thalamic structures, their connections, and ascending and descending fibers. Last, regarding proposes types of reactions to pain, the model proposes pain perception reactions, motor reactions, and stress reactions. These may vary and may have dramatic effects on subsequent pain trajectories.

Regarding pain mechanisms and phenotypes, pain has traditionally been characterized as either nociceptive or neuropathic. However, the prevalence of the phenotype of unexplained widespread chronic pain, not only in SCD but also in fibromyalgia (50), irritable bowel syndrome (51), and chronic pelvic pain (52), has forced authorities to adopt a third pain mechanism, nociplastic pain (53), distinct from nociceptive pain (i.e., pain due to local inflammation or tissue damage) and from neuropathic pain (i.e., pain due to nerve damage). Research on this mechanism is far less mature. Experts agree that nociplastic pain is not a distinct entity, but rather part of a chronic pain continuum. Nociplastic pain results from neuroplasticity, in this case, an acquired, augmentation of sensory and CNS signal processing, including pain perception and pain modulation. Initially called central sensitization (CS), to emphasize afferent pathways, CS is now known to involve activation or disinhibition of descending pain pathways as well (54), via activation of N-methyl-D-aspartate (NMDA) receptors. Indeed, significant cross talk exists between mu-opioid receptors and NMDA receptors. In SCD, CS is postulated to arise from years of repetitive vaso-occlusive, inflammatory, or other stimulation from SCD, leading to, a reset of the “pain thermostat” in the neuromatrix (55, 56).

Phenotypically, nociplastic pain produces quite variable symptoms, but typically amplified and (over)maintained clinical pain (57) including hyperalgesia—an increased sensitivity to a painful stimulus- or allodynia -pain that is provoked by a stimulus that is normally non-painful. Often, patients have widespread rather than localized pain (58). Widespread, bilateral pain is common in SCD (59). SCD patients with CS have more acute pain, worse sleep, and more psychosocial disturbances than others (60). Anatomically, nociplastic pain is associated with inflammation, not only in central neurons, but also in central glial cells (61, 62). But as stated, the triggers are unknown for transformation of the pain phenotype from intermittent acute, localized pain to widespread chronic pain, the “chronification” of pain.

Framing pain using the neuromatrix justifies treating pain in SCD using precision medicine and integrative health approaches. Already, research guided by this framework has been aimed at investigating the inputs, pain responses, phenotypes, and predictors of pain response in SCD and other pain disorders (63–70). Future research can be aimed at treatment that is more scientific, rather than empiric and atheoretic.

### 1.4. Article purpose

**Figure 2:**
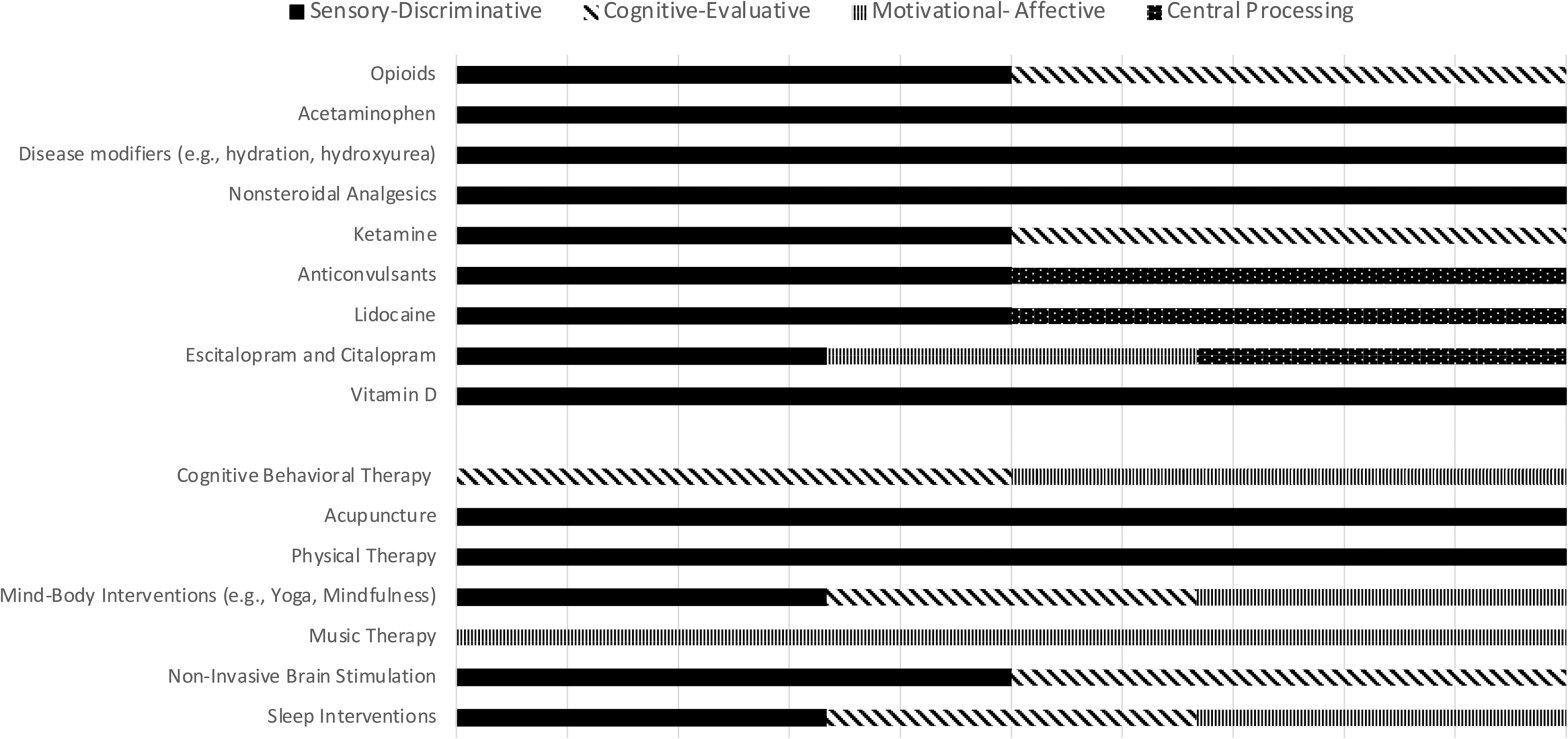
Standard and alternative sickle cell disease pain therapies, and their hypothesized target (s)—sensory-discriminative, cognitive-evaluative, motivational-affective, and pain processing—based on the neuromatrix model of pain. Multiple targets are hypothesized for many therapies.

Thus, this non-systemic topical review is intended to inform the development of precision medicine, integrative healthcare approaches to SCD pain. We have organized the various therapeutic approaches around the updated neuromatrix model. Figure 2 hypothesizes the efficacy of various currently used approaches using the neuromatrix model. The intent of this review is to give practitioners a theoretical basis for multimodal pain therapy to patients with SCD, as well as to suggest a research agenda for improving SCD pain therapy. Figure 2 categorizes all interventions based on their hypothesized neuromatrix operative targets (sensory-discriminative, cognitive-evaluative, motivational-affective inputs, or pain processing). It implies how clinicians might combine standard and alternative/complementary approaches to attack multiple pain targets and perhaps achieve greater pain reductions and/or greater opioid sparing. The review also discusses, for each pharmacotherapeutic approach, whether pharmacogenetics has advanced such that clinicians may more intelligently select or more precisely use that approach for pain in SCD.

## 2. Opioids

Opioid analgesics, especially mu (μ) receptor opioid agonists, have long been the mainstay of acute palliative therapy for SCD pain. (71). The clinical pharmacology of opioids for pain has been extensively reviewed (72). They are active in the peripheral nervous system, at the spinal cord dorsal horn, the brain stem, thalamus, and cortex, modifying sensory neurons both in the ascending pain transmission system and modifying parts of the descending inhibitory system in the spinal cord. Mu opioids, however, are associated with several adverse effects that often discourage their use, including some specific to the SCD population: rapid pharmacodynamic tolerance, the need for a higher mu opioid dose to maintain the analgesic effect (73); increased renal opioid clearance in SCD necessitating higher doses, (74, 75). physical dependence, i.e., opioid withdrawal on removal of opioids or administration of an antagonist; paradoxical pronociceptive effects or opioid-induced hyperalgesia (76); increased mortality (77); broad endocrine effects (78); GI complications, and (79); euphoria and craving leading to opioid use disorder (80, 81) and/or use of heroin (82). For these reasons, enthusiasm for the use of opioids has been limited to their role in treating acute rather than chronic pain (83–88). Further, the evidence of the efficacy of chronic opioid therapy (COT) beyond 12 weeks has been scant (89–91). On the other hand, the harms of opioids in SCD and specifically of COT in SCD compared to the harms in other groups with CNCP are small (92–94).

CDC revised its 2016 published guidelines on COT (95) in 2022 (96), by explicitly excluding chronic pain management and COT related to SCD in 2022. The American Society of Hematology recommended against the use of COT in SCD unless pain was refractory to multiple other treatment modalities. However this ASH recommendation was conditional, and based on very low certainty in the evidence about effects. Implementing these recommendations, including the spirit of the CDC guidelines, is fraught with difficulty. One must select individuals with SCD who should safely be prescribed a trial of COT. One must wean individuals off COT who have been deemed to fail this trial. And one must predict or detect opioid use disorder (OUD) in a population where pain may relent and recur and/or may be acute-on-chronic.

## 3. Acetaminophen

Acetaminophen (APAP) is a widely used oral analgesic and is primarily believed to exert its analgesic effect centrally via cyclooxygenase (COX) inhibition with no significant peripheral anti-inflammatory effects (97). Oral, suppository, and intravenous forms are available (98). Inter-patient variability in efficacy and hepatotoxicity are the main precision medicine issues associated with APAP treatment (99). The variability is attributed to genetic polymorphisms in the *CYP2E1, CYP1A2, CYP3A4*, *UGT, SULT and GST* genes. However, to date, the interaction between these genes has not been well delineated, limiting the role of pharmacogenetic biomarkers in the clinical management of APAP (100, 101).

## 4. Disease modifiers

Hydration (102), hydroxyurea (103, 104), prescription-grade L-Glutamine (105), voxelotor (106), crizanlizumab (107), red cell (simple or exchange) transfusion (108), and allogeneic bone marrow transplantation or cord blood transplantation (109) are the mainstays of remittive therapy or potentially curative therapy with the possibility of improved pain in SCD. Each are underutilized, and transplantation is still considered experimental by many. When taken regularly, hydroxyurea reduces acute chest syndrome, ED visits, hospitalizations, “strong” opioid use, hospital days, health care costs (110–112), and lengthens life (113), but does not statistically significantly reduce daily pain in most patients (114). Similarly, though they reduce annualized hospitalizations, neither L-glutamine nor crizanlizumab were approved based on improvement in daily pain. Voxelotor was approved based on improvements in total hemoglobin. Transfusions, when chronic, may decrease hospitalization rates (115–117), but are not generally used to reduce pain. Even therapy with curative intent does not uniformly eradicate chronic (likely central) pain in individuals with SCD, but rather aborts acute nociceptive pain (118–120).

## 5. Non-steroidal analgesics

Nonsteroidal anti-inflammatory drugs (NSAIDs), such as celecoxib, ibuprofen, flurbiprofen, and ketorolac (121), are widely used in management of SCD pain. These medications exert their effects by inhibiting cyclooxygenase (COX) enzymes and suppressing prostaglandin production (122). Notably, NSAIDs function independently of the opioid-mu receptor system, potentially reducing the amount of opioids required for pain (123, 124). However, to date, studies of NSAIDs for SCD pain have shown no significant reduction in the duration of VOCs or pain scores (125–128), nor have they demonstrated opioid sparing (129, 130). Among children, first-line therapy with IV ketorolac and IV fluids resulted in adequate resolution of pain (and avoidance of opioids) in 53% of VOCs (131). NSAIDs’ well-known potential gastrointestinal, cardiovascular, and renal adverse effects restrict their prolonged or chronic use (132).

Cyclooxygenase 2 (COX-2) inhibitors such as R-flurbiprofen and MRS2578 are being studied in SCD (133), and lipoxygenase (LOX) inhibitors are being studied in chronic pain (134), in hopes of achieving analgesic efficacy without the undesired gastrointestinal and renal adverse effects.

NSAIDs are metabolized primarily by the polymorphic *CYP2C9* gene. The Clinical Pharmacogenetics Implementation Consortium (135) published the following standardizing terms for clinical pharmacogenetic test results: poor metabolizer, intermediate metabolizer, normal metabolizer, rapid metabolizer, and ultrarapid metabolizer. Using these terms, individuals with *CYP2C9* poor or intermediate metabolizer phenotypes could have increased drug exposure, resulting in increased adverse effects from NSAIDs, because metabolism to inactive metabolites is defective in these patients. The CYP2C9*2 allele is associated with greater ibuprofen and diclofenac toxicity. Poor or intermediate *CPY2C9* metabolizers need to be started on lower-than-normal starting doses of NSAIDs to avoid potential ADRs, whereas patients who are normal *CYP2C9* metabolizers may require higher doses of NSAIDs to achieve effective pain relief (136).

## 6. Ketamine

Ketamine, a potent analgesic and phencyclidine analogue primarily used as anesthetic, acts as a competitive antagonist of the N-methyl-D-aspartate (NMDA) receptor (137, 138), potentially interfering with central sensitization (139, 140). It is available in various formulations, including intravenous oral, intramuscular, intrarectal or intranasal administrations. Notably, ketamine exhibits opioid sparing effects by interacting with several other binding sites, including mu-opioid receptors and cholinergic and muscarinic receptors (137). Ketamine has also demonstrated efficacy in treatment-resistant depression in recent decades (141). Low doses of ketamine have been used and reported in case studies of *VOC* and acute and chronic SCD pain (142), especially when pain is refractory to opioids (143), although the supporting evidence remains of low certainty (144). A systematic review of the pharmacogenomics of ketamine found that variants in the *CYP450*, *OPRM*, and *BDNF* genes alter the above-defined metabolizer status of ketamine (poor, intermediate, etc.) and therefore may someday be useful as biomarkers to guide ketamine treatment (141).

## 7. Anticonvulsants: gabapentin and pregabalin

The gabapentinoids (pregabalin and gabapentin), analogues of the neurotransmitter, gamma-aminobutyric acid (GABA), are considered the cornerstone of the pharmacological management of neuropathic pain. While the precise mechanism of action is uncertain (145), in rats, gabapentin and pregabalin are believed to decrease central sensitization by binding to the α_2_δ-1 subunit of voltage gated calcium channels in the dorsal root ganglia (146, 147). However, systematic reviews in humans report differing conclusions concerning gabapentinoid effectiveness in preventing chronic pain (148, 149). Adverse effects include dizziness, somnolence, dry mouth, fatigue, ataxia, blurred vision, peripheral edema, and weight gain. Gabapentinoids are substrates of drug transporters OCTN1 and OCT2 encoded by the *SLC22A2* and *SLC22A4* genes respectively. Polymorphisms in these genes are known to alter the activity of both transporters (150). Dosing should be adjusted in patients with renal disease due to the drugs’ renal excretion.

## 8. Lidocaine

Lidocaine, a local anesthetic and antiarrhythmic medication, is a well-established non-opioid analgesic for post-operative pain, and used as a second-line agent for neuropathic pain. It is available in systemic and transdermal formulations. It is believed to interact with multiple molecular targets including sodium channels, NMDA receptors and G-protein coupled receptors to exert its therapeutic effects (151–153). The evidence of its efficacy in SCD is sparse (154, 155). It is metabolized by *CYP3A4* (156). The clearance of lidocaine does not appear to be affected by pharmacogenomic variability when administered perioperatively as part of multimodal analgesia (157).

## 9. Escitalopram and citalopram

The selective serotonin reuptake inhibitors (SSRIs), citalopram and escitalopram, are not only widely used for treating major depression and anxiety disorders in SCD, but also anecdotally for SCD pain. The serotonin reuptake site (serotonin transporter), placed presynaptically on serotonergic nerve terminals, is the key regulator of synaptic serotonin levels in the central nervous system (158). Escitalopram has demonstrated effects on neuropathic pain. Citalopram and escitalopram are metabolized primarily by *CYP2C19* and, to a lesser extent, by *CYP3A4* and *CYP2D6* (159). Amitriptyline can provide some pain relief in certain chronic non-cancer pain conditions. However, we found no published evidence either supporting or refuting the use of citalopram in patients with SCD pain. A Cochrane review of antidepressants to treat chronic non-cancer pain found insufficient data for a meta-analysis (160). A review of adult treatment for neuropathic pain included a meta-analysis which was unconvincing for these drugs (161). No predictive algorithm is currently available for dosing these drugs for pain management (162).

## 10. Vitamin D

Vitamin D use among individuals with SCD who are often Vitamin D deficient has been reported to curb pain but studies did not measure opioid use (163, 164).

## 11. Other pharmacologic targets and systems

A recent review suggests various targets and systems of pain sensation which may generate therapeutic approaches that may be fruitful (165). These include pain signaling pathways, neurotransmitters, and analgesic receptors such as Ca^2+^/calmodulin-dependent protein kinase II (166, 167), protein kinase C delta (PKCδ) receptor on GABAergic neurons (168), nociceptin opioid receptor (169), and cannabinoid receptors (170, 171) which have shown promise in relieving pain in preclinical animal models (172, 173). Not yet tried widely in SCD is neuromodulation via direct anatomic or physiologic interruption of signaling pathways known to incite pain and discomfort (174). Another approach being investigated in sickle mice is to target pain prior vaso-occlusive or inflammatory nerve damage believed to lead to chronic pain (171, 175) and attempt to alter or repair it. Examples include targeting the nerve-regeneration associated gene, small proline-rich protein 1A (SPRR1A) (176, 177). In the clinical arena, the term regenerative pain medicine means harnessing the body’s own reparative capacity to treat pain (178).

## 12. Integrative medicine approaches

### 12.1. General use

There are few studies of non-pharmacological, complementary pain management approaches to address SCD pain, but most support the promise of these therapies. A recent systematic review of non-pharmacological treatments to reduce SCD pain during childhood found only 10 RCTs and quasi-experimental studies, and found most had significant methodological limitations (e.g., small samples, lack of follow-up data or control groups) (179). This lack of rigorous investigations is in contrast to research indicating the majority of children with SCD use some type of complementary technique to augment their existing treatment regimens (180) and that, when offered integrative approaches that include complementary techniques, over 70% of children with SCD and their families report the approaches to be acceptable and likely effective for treating SCD pain in adolescence.

### 12.2. Cognitive behavioral therapy for pain

The majority of the studies and most promising evidence is for cognitive behavioral therapy for pain (CBT-P) (179). CBT-P focuses on training people on a suite of cognitive and behavioral skills to alter the affective and cognitive mechanisms of pain. Common components include psychoeducation concerning the biopsychosocial model of pain, cognitive restructuring, relaxation, and distraction. In the previous review, 5 of the 10 identified studies were focused on CBT-P, with overall findings indicating reductions in pain frequency and intensity. Findings also found no evidence that CBT-P reduced health care use due to pain, and most studies were small in size. Notably, there is an ongoing clinical trial examining the efficacy of CBT-P (181).

### 12.3. Mind-body interventions

Of the other promising treatments in the above review, biofeedback, virtual reality, and yoga each had only 1 study finding reductions in pain. Biofeedback was related to reduced analgesic use in SCD. Mindfulness meditation is being studied currently (182).

### 12.4. Acupuncture

A review of the use of acupuncture for pain management in pediatric SCD patients (183) found 5 studies with sample sizes ranging from one to 31. Overall, studies indicated that the treatment was feasible, acceptable, and an effective adjunctive pain treatment. These findings are consistent with findings from adult SCD studies indicating acupuncture reduces pain intensity and pain interference, including during acute SCD pain crises (184–187). However, these studies had small sample sizes, ranging from 6 to 47 patients. The primary methods were case reports and retrospective chart reviews, and there are no published RCTs. One ongoing study is testing guided relaxation and acupuncture for SCD pain in adults (188).

### 12.5. Physical therapy

A pilot RCT of 10 adults with SCD indicated conventional physical therapy and aquatic physical therapy were acceptable and lead to reduced SCD pain (189), and a 2-arm non-randomized clinical trial indicated a home-based therapeutic exercise program led to reductions in SCD pain (190).

### 12.6. Music therapy, non-invasive brain stimulation, sleep-based approaches

A mixed-method RCT of 24 adults with SCD found that music therapy led to reductions in pain intensity and pain interference (191). We found no published clinical trials of non-invasive brain stimulation or sleep-based interventions, such as CBT for insomnia.

## 13. Summary

Most of the components available for the assembly of a pain treatment plan for individuals with SCD, even when guideline-recommended, have little evidence for their recommendation. In this way, SCD is similar to other diseases which comprise chronic non-cancer pain. However, precision medicine and integrative health offer hope and a research agenda for a thoughtful multimodal, individualized approach to diagnosis and treatment of acute and chronic pain in SCD. They suggest the use of emerging tools, such as survey instruments, sleep evaluation, pharmacogenomic testing, and functional magnetic resonance imaging, in developing robust individual pain profiles/subphenotypes. They suggest the incorporation of multimodal pharmacologic and non-pharmacologic components into an individualized treatment plan, based on the diagnosed subphenotype of a given patient. And they each demand clinicians identify and avoid drugs or other therapies with an increased risk of causing adverse effects, drugs with a narrow therapeutic index, or drugs or other therapies predicted not to be efficacious (192) for individuals living with SCD.
